# An Automated, Quantitative, and Multiplexed Assay Suitable for Point-of-Care Hepatitis B Virus Diagnostics

**DOI:** 10.1038/s41598-019-52147-z

**Published:** 2019-10-30

**Authors:** Adi Wijaya Gani, Wei Wei, Run-Zhang Shi, Elaine Ng, Mindie Nguyen, Mei-Sze Chua, Samuel So, Shan X. Wang

**Affiliations:** 1 Department of Electrical Engineering, Stanford University, Stanford, California 94305 USA; 2 Asian Liver Center, Department of Surgery, School of Medicine, Stanford University, Stanford, California 94305 USA; 3 Department of Pathology, Stanford University School of Medicine, Stanford University, Stanford, California 94305 USA; 4 Department of Materials Science and Engineering, Stanford University, Stanford, California 94305 USA; 5 Department of Medicine, Stanford University School of Medicine, Stanford University, Stanford, California 94305 USA

**Keywords:** Biosensors, Assay systems

## Abstract

Hepatitis B virus (HBV) infection has a global reach with high prevalence in resource-limited areas like China and Africa. HBV patients in these areas have limited access to the currently used, costly HBV assays, which are performed in centralized clinical laboratories using single-plexed assays with bulky and expensive instruments. We aim to overcome these limitations by developing a simple and affordable HBV diagnostic platform to allow for timelier diagnosis and intervention of HBV infection. Using giant magnetoresistive (GMR) biosensor chips, we developed an automated and multiplexed quantitative platform for the measurement of a panel of HBV serology markers, including HBV “e” antigen (HBeAg), HBV surface antigen (HBsAg), and the antibody against HBsAg (anti-HBs). Our assay platform was able to detect each HBV marker with high specificity and sensitivity (with three orders of magnitude in dynamic range for each marker). Blinded analysis of HBV patient sera showed excellent correlation between our multiplexed quantitative HBsAg results and the qualitative results obtained using FDA-approved immunoassays, as well as those obtained using quantitative, single-plexed, enzyme-linked immunosorbent assays (ELISAs). The portable, automated, multiplexed, quantitative HBV serology assay platform we designed shows great promise as a more accessible alternative for HBV screening, diagnosis, and treatment monitoring.

## Introduction

To increase the sensitivity and accuracy of diagnostic assays, it is important to be able to detect low-abundance protein biomarkers in blood. A multistep sandwich immunoassay is typically the assay system of choice, given that it incorporates stepwise incubation and washing, and thus helps reduce the background signal caused by non-specifically-bound proteins in blood. These multistep immunoassays are performed in clinical diagnostic laboratories by automated chemiluminescence-based assays utilizing paramagnetic particles. This approach is expensive and requires trained technicians and dedicated instruments that are bulky and immobile. These factors prevent the use of such assays outside of central laboratory settings and limit the accessibility of patients in low-income and rural areas. In our work, we developed a portable, automated, multiplexed immunoassay that can simultaneously detect low-abundance HBV infection serology biomarkers using the giant magnetoresistive (GMR) biosensor platform. Automation was accomplished using an electromechanical fluidic system controlled by a microcontroller. This system can be readily deployed in point-of-care (POC) settings and can be operated by personnel with minimum training. The rapid availability of test results at POC would significantly improve health-care delivery in those areas. We chose HBV infection as our disease of focus, as it is a health-care problem with a global reach, particularly affecting people in developing countries where access to costly and sophisticated diagnostics is limited.

Chronic HBV infection is among the leading causes of preventable death^1,2^. It is estimated that 260 million people worldwide are living with chronic HBV infection. These individuals have a significantly increased risk of developing chronic liver diseases and liver cancer compared to the general population. The spread of HBV infection could be greatly reduced with timely screening, diagnosis, monitoring, antiviral treatment, and vaccination^3,4^. Currently, the prevalence of chronic HBV infection is underreported because many patients remain unidentified due to large deficiencies in HBV awareness and screening. This leaves many treatment-eligible patients untreated, thus further increasing morbidity and mortality rates related to HBV and hepatocellular carcinoma (HCC). The poor rate of screening and the resulting poor linkage to care may be due, in part, to limited access to affordable and convenient testing^5^. Viewed as a preventable disease, the World Health Organization (WHO) aims to eliminate viral hepatitis by 2030 by reducing new chronic infections by 90% and the mortality rate by 65%^6,7^. The achievement of these goals can be expedited by the availability of rapid and inexpensive HBV screening and diagnostic assays at POC.

In many parts of the world, the rapid strip test, based on lateral flow immunoassay, is currently the most commonly used HBV POC diagnostic^8,9^. These assay results are qualitative in nature, generating binary “positive” or “negative” readouts and are inadequate for monitoring the infection status or treatment efficacy over time. Within the past 10 years, two meta-analyses were performed to assess the diagnostic accuracy of rapid diagnostic tests for HBV surface antigen (HBsAg) detection^10,11^. Both analyses reported high pooled sensitivity and specificity which makes them valuable for HBV diagnosis in resource limited areas. However, substantial heterogeneity was observed between brands, as well as within the same brands. The rapid diagnostic tests evaluated were all qualitative in nature (such as Determine HBsAg, BinaxNow HBsAg); moreover, these tests had analytical sensitivity higher than what was claimed by the manufacturers, had reduced sensitivity with HBsAg mutants, and performed poorly in seroconversion panels. Pooled sensitivity of these qualitative rapid HBsAg tests were also reported to be lower in individuals co-infected with HIV. Rapid tests for HBV “e” antigen (HBeAg) and antibody against HBsAg (anti-HBs) are in different stages of development and clinical applications, and are all qualitative in nature^12–14^. In addition, a recent performance study of commercially available rapid tests for anti-HBs showed insufficient sensitivity^12^.- The quantitative POC HBV diagnostic technology that we aim to develop offers the advantages of multiplexing several HBV biomarker assays to provide simultaneous, rapid, and sensitive measurements of these biomarkers. This collective information can be used to accurately diagnose the status of an individual’s HBV infection (active or inactive carrier), and importantly, allow the monitoring of a patient’s response to treatment to ensure that patients are connected to appropriate medical care for long-term monitoring and antiviral treatment.

As a proof of concept of our automated assay system using the GMR biosensor platform, we developed a multiplexed quantitative assay to detect three HBV infection biomarkers: HBeAg, HBsAg, and anti-HBs. Multiplexed assays of the three markers can provide preliminary screening and diagnosis of HBV infection and data regarding the status and severity of disease^15^. Quantitative HBsAg measurement determines whether a person has a chronic infection or is likely to be an inactive carrier. The detection of HBeAg in individuals not receiving antiviral treatment is generally indicative of high viral replication and infectivity associated with a high HBV DNA load. Quantitative HBeAg testing can be a cost-effective alternative to the expensive HBV DNA viral load test, which currently can only be performed in a central laboratory. Quantitative and serial measurements of HBsAg and HBeAg combined can be used to assess a patient’s response to antiviral drug treatments^16,17^. For patients undergoing antiviral treatment, such as pegylated interferon therapy, an informed decision to stop treatment in non-responders can be highly cost-saving^18^. Quantitative anti-HBs testing can help determine whether an individual is protected against future HBV infection; in the United States, an individual who has an anti-HBs level of 10 mIU/ml or higher is considered immune against future HBV infection^19^.

In the regional and global strategies to eliminate mother to child transmission of HBV, HIV, and syphilis (triple elimination), all pregnant women are encouraged to have early antenatal HBsAg testing^20^. A rapid test consisting of HBsAg and HBeAg has great value in antenatal screening and in the prevention of mother-to-child transmission of HBV, which is one of the major routes of transmission. HBeAg levels would provide additional information to identify women who are likely to have high viral load and would benefit from prophylactic tenofovir treatment in her third trimester to prevent the baby from becoming infected^21^. Newborns to HBsAg-positive and/or HBeAg-positive mothers are at increased risk of perinatal transmission; therefore, accurate identification of these mothers and newborn will guide clinical management to reduce mother-to-child transmission. The combined quantitative measurement of these three HBV markers at POC settings offers a valuable means to detect and monitor HBV infection, and to reduce HBV transmission.

## Results

### Correlation of automated GMR multiplexed HBV assays with qualitative commercial assays

**Figure 1:**
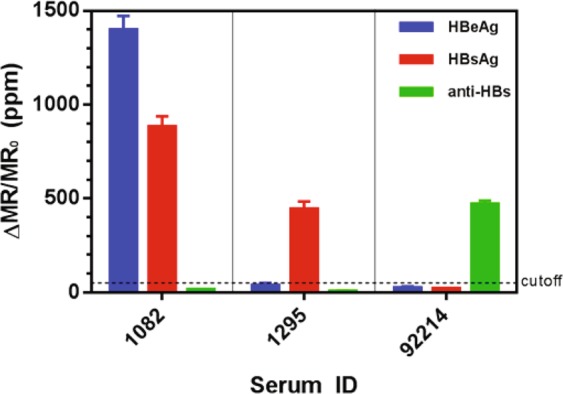
Automated GMR three-plex assays measurement results. Sera 1082 and 1295 were from HBV patients, and serum 92215 was from a healthy individual. HBV sera have either HBeAg or HBsAg but no anti-HBs. Healthy sera do not have HBeAg or HBsAg but may have anti-HBs (e.g., from vaccination).

As a first step in validating the diagnostic ability of our automated, multiplexed HBV biomarker assay, we tested HBeAg, HBsAg, and anti-HBs levels in one healthy serum (92214) and two HBV-infected sera (1082 and 1295) (Fig. 1). Our assay accurately diagnosed these samples, showing that serum 1082 was positive for both HBeAg and HBsAg, and that serum 1295 was positive for HBsAg only (Fig. 1). The healthy serum sample 92214 was negative for both HBeAg and HBsAg but positive for anti-HBs, consistent with HBV-free, post-vaccination status.

We next performed a blinded analysis of 10 HBV-infected patient sera to qualitatively categorize the samples using our GMR multiplexed HBV assays. Threshold signal values (or cut-off points) were determined, as described in the Methods section. Briefly, the threshold signal value for each GMR-based assay was set at the zero-analyte signal plus two standard deviations. For the HBsAg and HBeAg assays, samples with signals below the threshold were qualitatively diagnosed as healthy, whereas samples with signals above the threshold were qualitatively diagnosed as diseased. For the anti-HBs assay, samples with signals below the threshold were diagnosed as diseased, whereas samples with signals above the threshold were qualitatively diagnosed as healthy. We observed consistent agreement between our qualitative HBsAg results and the qualitative results obtained using FDA-approved immunoassays (Architect 1500, Abbott Diagnostics, Libertyville, IL) at Stanford Hospital Clinical Laboratory, as shown in Table 1. Three patients (P1–P3) were HBeAg positive, and nine patients (P1–P9) were HBsAg positive. Patient P10 was HBeAg and HBsAg negative. A review of the patients’ medical charts revealed that patient P10 had a history of HBV infection, and was given anti-viral treatment. The blood sample obtained from patient P10 was at a time point after successful anti-viral treatment. As expected, all of the tested HBV patients had undetectable anti-HBs in their sera, since the antibody would have bound and formed a complex with HBsAg^22^.

**Table 1:** Comparison between qualitative GMR assay results and qualitative results obtained from the Stanford Hospital Clinical Lab.

	Sample ID	HBsAg		HBeAg		Anti-HBs	
		GMR	Stanford Hospital Clinical Lab	GMR	Stanford Hospital Clinical Lab	GMR	Stanford Hospital Clinical Lab
HBV	P1	+	+	+	+	−	−
	P2	+	+	+	+	−	−
	P3	+	+	+	+	−	−
	P4	+	+	−	−	−	−
	P5	+	+	−	−	−	−
	P6	+	+	−	−	−	−
	P7	+	+	−	−	−	−
	P8	+	+	−	−	−	−
	P9	+	+	−	−	−	−
	P10	−	−	−	−	−	−

### Quantification by automated GMR multiplexed HBV assays is consistent with single-plexed quantitative commercial assays

We also compared our GMR results with other quantitative immunoassay tests: (1) HBsAg and HBeAg using ELISA; (2) anti-HBsAg using chemiluminescent microparticle immunoassay (CMIA) on the commercial Abbott Architect at Stanford Hospital Clinical Laboratory; and (3). HBsAg using the Abbott Architect platform at Quest Diagnostics. We tested 10 HBV-infected patients (P1-P10), and five individuals with no history of HBV infection as controls. The samples were de-identified when the GMR assay was performed.

**Figure 2:**
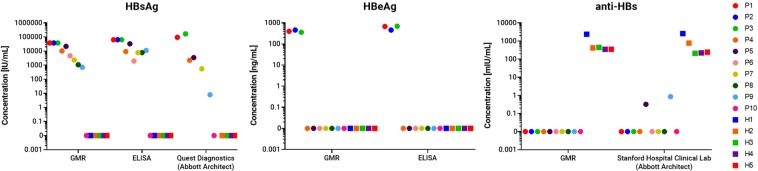
Quantitative comparisons between the serum measurement results obtained with the automated GMR biosensor system and other diagnostic methods (ELISA and/or Abbott Architect). Each colored dot represents a single patient’s analyte concentration obtained across the different platforms. Healthy sera (squares) do not have HBeAg or HBsAg but may have anti-HBs (e.g., from vaccination). HBV sera (circles) have either HBeAg or HBsAg but do not have free anti-HBs. The measurement results, done with the GMR, ELISA, and Abbott Architect, are consistent and show strong correlation.

Figure 2 shows the comparison of the test results obtained by the automated three-plex GMR biosensor system and other quantitative immune-based diagnostic methods. Consistent across different assay platforms, all five healthy sera tested negative for HBeAg and HBsAg, and had anti-HBs levels that were adequate (>10 mIU/ml) for protection against HBV infection. Both GMR and Abbott anti-HBs assays consistently showed the absence (or extremely low levels) of the free anti-HBs in all 10 HBV-infected samples. The absolute concentrations of HBeAg and HBsAg measured showed strong concordance between the GMR and ELISA assays in all 10 HBV-infected samples.

Of the 10 HBV-infected samples, six (with sufficient volume) were sent out to a national reference laboratory (Quest Diagnostics Inc., Secaucus, NJ)^23^, to provide further independent validation of our quantitative HBsAg assay. Table 2 shows the concentration levels of all three analytes as quantified by the various detection methods. The sensitivities and specificities, along with their respective 95% confidence intervals (CIs) were also calculated and shown in Table 2. Our GMR assay demonstrated similar or better sensitivities and specificities than current conventional methods. When the Bland-Altman^24^ and linear regression analyses were done to compare GMR results with those obtained by other conventional methods, we observed good correlation between different assay platforms (within 95% limits of agreement for Bland-Altman, and R^2^ > 0.9 for linear regression) (Supplementary Figs 1 and 2). Taken together, these results provide validation that our automated multiplex GMR assay performs equally well compared to other quantitative assays, especially the assays performed on the Abbott Architect platform (for quantitative HBsAg and anti-HBs).

**Table 2:** Comparison between quantitative GMR assay results and quantitative results obtained from gold standard ELISA and Abbott Architect at commercial clinics.

	Sample ID	HBsAg [IU/mL]			HBeAg [ng/mL]		Anti-HBs [mIU/mL]	
		GMR	ELISA	Quest Diagnostics (Abbott Architect)	GMR	ELISA	GMR	Stanford Hospital Clinical Lab (Abbott Architect)
HBV	P1	>38000	>63840	91590	400	672	0	0
	P2	>38000	>63840	N/A	460	460	0	0
	P3	>38000	>63840	166300	360	695	0	0
	P4	10252	9245	2220	0	0	0	0
	P5	21664	32940	3460	0	0	0	0.3
	P6	4644	1961	N/A	0	0	0	0
	P7	2371	7742	550	0	0	0	0
	P8	1083	7781	N/A	0	0	0	0
	P9	711	11128	8	0	0	0	0.9
	P10	0	0	0	0	0	0	0
Healthy	H1	0	0	N/A	0	0	2380	2585
	H2	0	0	0	0	0	420	763
	H3	0	0	0	0	0	440	208.2
	H4	0	0	0	0	0	345	220.6
	H5	0	0	0	0	0	350	242.6
	Sensitivity (95% CI)	90% (60–99%)	90% (60–99%)	70% (40–89%)	90% (60–99%)	90% (60–99%)	100% (72–100%)	100% (72–100%)
	Specificity (95% CI)	100% (57–100%)	100% (57–100%)	100% (57–100%)	100% (57–100%)	100% (57–100%)	100% (57–100%)	100% (57–100%)

## Discussion

The rapid strip test is widely used at POC worldwide, but it is qualitative and has limited accuracy. A quantitative hepatitis B serologic test has the advantage of providing valuable clinical information for long-term monitoring of chronic hepatitis B infection. In this study, we performed a proof-of-concept study using our automated assay device, based on GMR sensor technology, for the multiplexed detection of three HBV biomarkers: HBeAg, HBsAg, and anti-HBs. Used together, these three biomarker levels can provide information on diagnosis, severity of the infection, efficacy of a viral treatment, and the immunity status against HBV infection. Automation of the typical multistep sandwich immunoassay was achieved by a prototype electromechanical fluidic system controlled by a microcontroller. This device is suitable for use at POC by personnel with minimum laboratory training, thus increasing access to diagnoses and care of HBV patients in resource-limited areas where HBV may be prevalent.

The results obtained from our automated multiplexed assay device were consistent with the biomarker levels obtained through independent immunoassays for HBeAg, HBsAg, and anti-HBs levels in both HBV-infected patients and healthy individuals. Our assay was able to accurately diagnose the HBV-infection status of individuals, while identifying those who are HBV-free. Among the 10 HBV-infected patients studied, only three patients (P1, P2, and P3) had non-zero levels of HBeAg; these three patients also had the highest concentrations of HBsAg ( > 38000 IU/ml). Since HBeAg is a biomarker of active viral replication in the infected host, the presence of HBeAg might correlate with high concentrations of HBsAg in the host. Importantly, the quantitative HBsAg data from our automated assay highly correlate with that obtained from a certified clinical diagnostic laboratory, suggesting that our prototype assay has the potential to offer a cheaper and more accessible diagnostic test than those currently used (which are expensive and inaccessible to many HBV patients in rural areas). Both qualitatively and quantitatively, our results provided the same diagnosis for the tested samples. Our GMR-assay reported one false negative diagnosis (in P10). However, this false negative was consistently reported on the Abbott Architect platform. The false negative result can be attributed to reasons previously described in the Results section. We noted discrepancies between the absolute quantitative HBsAg values obtained by the GMR assay and the Quest Diagnostics assay, which may be caused by the different HBsAg calibration standards used in the two tests.

Our automated device embodies several advantages that characterize a good POC device, thereby enabling its potential to help advance health care in developing countries or resource-limited areas. Firstly, it is lightweight, compact, and portable. Users can bring it almost anywhere and use it anytime. Secondly, it is easy to use, designed to be user-friendly and intuitive. User interaction is minimized to a simple finger prick and pressing of the “Start” button on the smartphone. Automation of the device also minimizes user error. Such a design eliminates the need for a trained operating professional. Thirdly, it provides relatively quick results, taking about one hour to complete the assay. We envision that refinements to the assay will further reduce the time taken. Fourthly, there is minimal need for constant calibration of the device, as the quality of the assay is highly dependent on the GMR biosensor chip itself and the biochemical reagents. Therefore, calibration can be done similarly to running an assay on the device, but by using a solution of standard analytes with predetermined concentrations in place of blood sample. Day-to-day results from running the assay on this standard solution on the device should remain consistent. Lastly, our device is inexpensive relative to current diagnostic lab tests. As seen in the cost analysis (Tables 3 and 4), the one-time purchase of our automated device is $156, which is significantly cheaper than the $130,000 Abbott Architect used in commercial labs, such as Quest Diagnostics. Because our device uses very little reagent and the GMR biochips are inexpensively mass produced, the recurring cost per HBV diagnostic test is $5.75 (for three HBV biomarkers). This is also significantly cheaper than the recurring cost of approximately $540 for the same HBV diagnostic test at commercial labs. The advantages provided by our automated POC device makes it a practical alternative for use in resource-limited settings. In comparison to the currently available technology for HBV detection, our GMR sensor arrays exhibit larger dynamic range, lower limits of detection, comparable or smaller sample volume, and higher multiplexing capabilities (Table 5). In addition, it can be made POC and is cheaper than the traditional ELISA and Abbott Architect chemiluminescence assay. Although the GMR sensor arrays are more expensive than the lateral flow assay as a POC device, it provides more accurate and sensitive quantitative results.

**Table 3:** GMR platform cost analysis.

GMR System	
Parts	Cost [$]
Microcontroller (Arduino)	20.00
PCB and electrical parts	20.00
Peristaltic pumps	50.00
Rechargeable Lithium Polymer (LiPo) Battery	16.00
3D-printed case	50.00
Automated Device Cost (One-time Cost)	156.00
Consumables	Cost [$]
GMR biochip	1.00
Cartridge	0.25
Magnetic nanoparticles	2.50
Reagents (three assays)	2.00
Cost per HBV test (Recurring Cost)	5.75

**Table 4:** Abbott Architect (commercial lab) platform cost analysis.

Commercial Lab (e.g. Quest Diagnostics)	
Machine	Cost [$]
Abbott Architect	>100,000.00
Machine Cost (One-time Cost)	>100,000.00
Assay	Cost [$]
HBsAg (quantitative)	400.00
HBeAg (qualitative only)	70.00
Anti-HBs (quantitative)	70.00
Cost per HBV test (Recurring Cost)	540

Assay costs for HBsAg, HBeAg, and anti-HBs are provided by Quest Diagnostics, and are inclusive of reagents and labor.

**Table 5:** Comparison of our GMR sensor array platform with other current detection methods.

Technology	Detection Method	Dynamic Range (log)	Limit of Detection	Sample Volume	Multiplex Capability	Quantitative Results	Point-of-Care	Recurring Cost (Per Test)
GMR Sensor Array	GMR	3–4	1 ng/mL	5–25 uL	5–10-plex	Yes	Yes	$
ELISA	Colormetric	2–3	1 pg/mL–1 ng/mL	50–100 uL	1-plex	Yes	No	$$
Abbott Architect	Chemi-luminescence	3–4	1–10 pg/mL	10–150 uL	1-plex	Yes	No	$$$
Lateral Flow Assay	Colormetric	—	100 ng/mL	50 uL	2–4-plex	No	Yes	$

In addition to HBsAg, HBeAg, and anti-HBs, there have been growing interest in the use of other biomarkers for HBV detection, including HBV viral load, alanine aminotransferase (ALT), and hepatitis B core-related antigen (HBcrAg). Given that ALT and HBcrAg are both protein biomarkers, they can be easily incorporated into our current HBV protein biomarker panel. HBV viral load is determined by the HBV DNA level. We have previously shown the feasibility of detecting DNA on our GMR biochips^25–27^. The incorporation of an HBV viral load assay is therefore feasible on our platform, although considerable modifications in the machine design will be needed to allow for temperature cycling for DNA amplification steps, and to ensure that this does not interfere with the temperature-sensitive protein-based immunoassay. The automation of such a multi-plex assay is expected to enhance the diagnostic value of the device.

As a next step, the current prototype assay device could be further miniaturized by using mini diaphragm pumps and by integrating the control circuitry board with the GMR readout circuit board. A longitudinal study with a larger number of patients in various resource-limited settings is necessary to further evaluate the clinical utility of the device and to collect practical user feedback. With a larger patient cohort, we also expect that the strength of the correlation between our assay and conventional methods to be improved. Lyophilization (freeze drying) of reagents will be used during field implementation, which is expected to greatly extend the heat-stability and shelf life of heat-sensitive chemicals. In addition, the automated device uses off-the-shelf simple circuitry, battery, and mechanical components that are sold in many parts of the world with widely varying climates. We anticipate that the device should be able to cover a wide range of operating temperatures.

Our pilot study involves a small number of HBV-infected individuals, of whom all are Asians. While our results are encouraging, it would need further validation across variable HBV genotypes to ensure its universal use; for example, in Asian patients with genotypes B and C, and in African patients with genotypes E (in the West), A (in the South), and D (in the North). A correlation study between the quantitative HBsAg levels measured by our assay, and HBV DNA levels would further strengthen its clinical value and add clinical perspective to its use. This assay platform and the GMR sensor technology could be adapted to detect various protein biomarkers in different disease areas, and therefore, it has significant applicability in clinical diagnostics. Fundamental to the success of this assay is a pair of high-performance capture and detection antibodies or antigens for the biomarkers of interest. The compartmentalized cartridge allows for the simultaneous detection of both antigens and antibodies from a single sample.

The availability of a quantitative immunoassay POC device that is easy to use and highly accurate could replace the popular lateral flow tests, which are less sensitive and accurate and provide only qualitative results. With accurate quantitative diagnostics, more descriptive information about the health status of the users could be gleaned, and more informed medical decisions could be made. This is especially important for diseases like HBV, which can be controlled with timely diagnosis, treatment, and monitoring to greatly reduce morbidity and mortality that are not only associated with HBV infection itself but also with chronic liver diseases and liver cancer, which are highly associated with HBV infection. The widespread availability of affordable, accurate, quantitative diagnostics will be an important step in eliminating the global burden of preventable diseases. The process of further development, miniaturization, and validation of this type of innovative quantitative diagnostic platform has significant potential and value for increasing access to health care and providing timely medical diagnoses.

## Methods

### GMR biosensor

The GMR sensor chips were fabricated as described in other studies^28,29^. Briefly, the chip has dimensions of 1.2 cm × 1 cm, with an array of 80 (8 × 10) GMR spin valve sensors. Each sensor occupies an area of 100 µm × 100 µm. In our study, in-plane spin valve sensors were deposited on Si/SiO_2_ substrate. The compositions and thicknesses (in nm) of each layer from bottom to top were as follows: Ta (3)/seed layer (4)/PtMn (15)/CoFe (2)/Ru (0.85)/CoFe (2)/Cu (2.3)/CoFe (2)/Cu (1)/Ta (4). The structure was passivated with SiO_2_ (10)/Si_3_N_4_ (20)/SiO_2_ (10) to protect the sensors from direct exposure to the fluids.

### Capture probe immobilization

The GMR biosensor chip was surface-functionalized with (3-aminopropyl) triethoxysilane (APTES)-like surface chemistry. The chip was spotted with droplets of HBeAg and HBsAg capture antibodies, as well as HBsAg capture protein using a robotic nanopipette (Scienion AG, Berlin, Germany). Each droplet (1.5 nL) covered one individual GMR sensor. Bovine serum albumin (BSA) was spotted as a negative control. The prepared chip was incubated overnight at 4 °C in a humidity chamber. Subsequently, the sensor chip was washed and blocked with 2% BSA in the phosphate-buffered saline (PBS) solution for one hour. After blocking, it was washed and dried before use.

### HBV assay designs and standard curves

**Figure 3:**
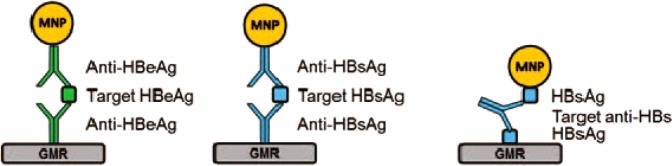
HBeAg, HBsAg, and anti-HBs sandwich magnetic immunoassay structures.

We developed three bioassays (HBeAg, HBsAg, and anti-HBs) to measure HBV serum biomarker concentrations (Fig. 3). For the antigen assays (HBeAg and HBsAg), a forward-phase, sandwich-type immunoassay was used. A capture antibody specific to the target antigen was immobilized on the GMR biosensor. Next, the target antigen was captured and immobilized by the capture antibody. After the incubation and washing steps, a detection antibody specific to the target antigen was delivered to the reaction well. The detection antibody was biotinylated to capture free-floating, streptavidin-coated magnetic nanoparticles (MNP) onto the GMR sensor.

For the anti-HBs antibody assay, the HBsAg protein (inclusive of all subtypes) was immobilized on the GMR sensor. If a sample solution contains the anti-HBs antibody, the antibody will bind to the HBsAg capture probe on the sensor. After a washing step, to complete the assay, a biotinylated HBsAg antigen (all subtypes included) was delivered to the reaction well. This allowed the streptavidin-coated MNPs to bind to it, forming the HBsAg-antibody-HBs complex. The binding of HBsAg antigens to both antigen binding sites of the anti-HBsAg antibody ensures that only the free anti-HBs antibody was captured and detected by the assay. Most patients with HBV infection do not have the free anti-HBs antibody in their blood^30,31^. On the other hand, healthy individuals who have immune protection against HBV infections will have the free anti-HBs antibody in their blood.

**Figure 4:**
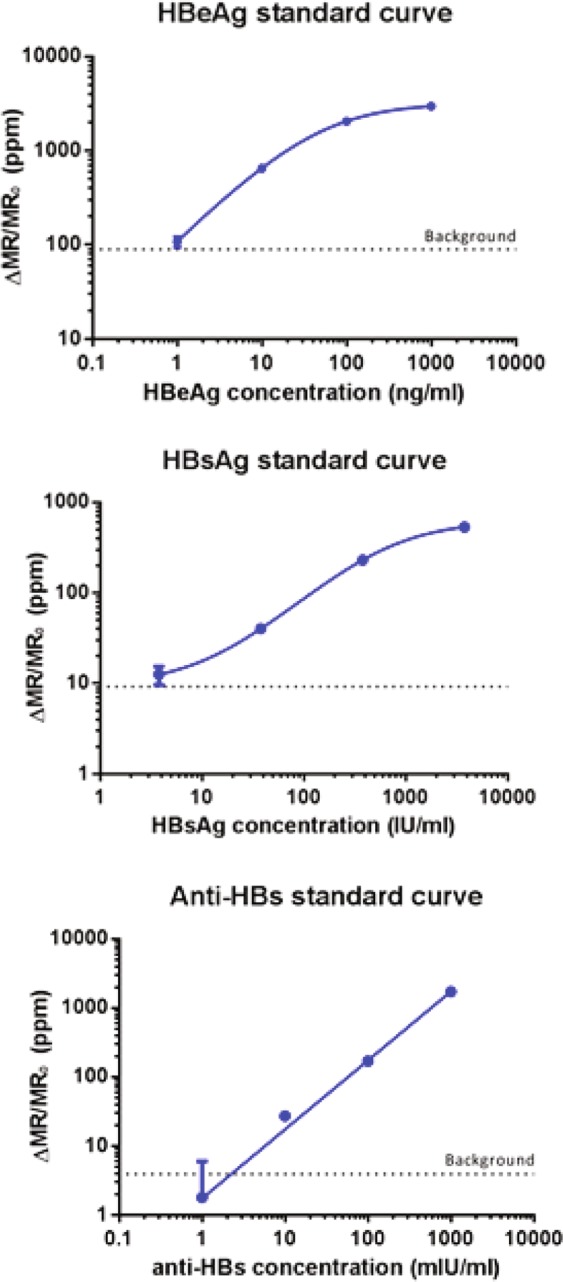
HBeAg, HBsAg, and anti-HBs assay standard curves obtained with the automated GMR biosensor system.

The standard curves of the assay are shown in Fig. 4. The GMR sensor signals (∆MR/MR_0_), derived from change of the GMR resistance due to the MNP labels normalized by its initial magnetoresistance (MR), and expressed in parts per million (ppm), are shown on the y-axes. The concentrations of the target analytes are shown on the x-axes. The dotted lines represent the cut-off points, which is defined as negative control sensor signals (zero analyte) plus two standard deviations. GMR sensors conjugated and covered with bovine serum albumin were used as the negative control. These cut-off points indicate the sensitivity or the lower limit of detection (LOD) of the assay. The curve fitting equations were chosen based on best fit. For the anti-HBs assay, the hyperbolic fit was used. For the HBsAg and HBeAg assays, the 4PL fit was used. We calculated the analyte concentrations from the curve fit equations. By serially diluting the analytes, the HBeAg assay was tested to have a LOD of 1 ng/ml (approximately 1 IU/ml)^32^, the HBsAg assay has a LOD of 3 IU/ml, whereas the anti-HBs antibody assay has a LOD of 1 mIU/ml. Conversion factors from IU/mL to ng/mL were provided by the World Health Organization (WHO). All three assays have three orders of magnitude in dynamic range.

### Assay compartmentalization

**Figure 5:**
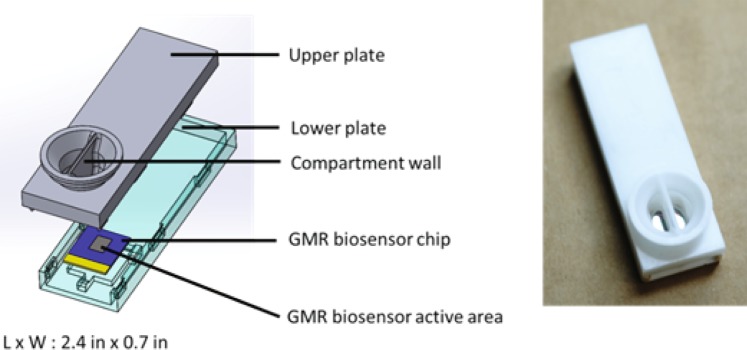
The custom-designed cartridge with a barrier wall in the middle of the well, creating two separate compartments to separate the two antigen assays from the antibody assay.

To avoid cross-reaction between the HBsAg and anti-HBs antibody assays, the assays were performed in separate compartments. The two antigens assays (HBsAg and HBeAg) were performed in the same compartment. We designed a custom cartridge that had one reaction well, separated by a barrier wall, thus creating two separate compartments (Fig. 5). The cartridge material used was Delrin, an acetal homopolymer resin thermoplastic that is strong, watertight, and biocompatible.

### Automated assay device

We built a prototype of an automated but simple POC bioassay device as proof of concept. The goal of the automated assay device is for an end user to be able to perform HBV testing with just a one-touch input after the samples have been delivered into the reaction well, followed by pre-programmed incubation, washing, and reagent delivery.

**Figure 6:**
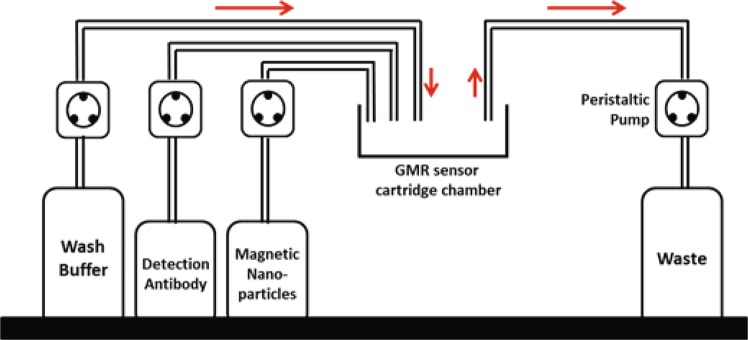
Schematic of the automated assay system. Three peristaltic pumps ensure that the wash buffer, detection antibody, and magnetic nanoparticle flow separately into the GMR cartridge. After usage, the liquid waste is transported out of the reaction well to the waste container through another peristaltic pump.

The automated assay device (Fig. 6) uses four peristaltic pumps (Adafruit Industries, New York City, NY) to move the reagents and deliver the wash buffer, detection antibodies, and MNPs to the cartridge reaction well, as pre-programmed. One pump is used to drain the reagents from the cartridge chamber to a waste container. The red arrows indicate the directions of the reagent flow from the reservoir containers to the reaction well in the cartridge and, finally, to the waste container, with a flow rate of 27 ml/min.

A pump driver circuit and an Arduino microcontroller (Supplementary Fig. 3) are used to control the pumps; to select the pump to be on; and for the duration, as well as the sequence, of reagent delivery from the storage containers to the cartridge reaction well and, finally, to the waste container. The entire device is powered by a LiPo 7.4-volt portable battery.

### Enzyme-linked immunosorbent assay (ELISA) protocol

The ELISA plate (Nunc Maxisorp Microplates, VWR, Radnor, PA) was coated with the capture antibody (4 μg/ml in PBS, 100 μl/well) overnight, followed by blocking for 2 h with 1% BSA in PBS (200 µl/well). After three washing steps with PBS-T, the wells were incubated for 1 h with 100 μl/well of serum diluted in 1% BSA in PBS containing. After the washing step, the wells were incubated for 1 h with 100 μl/well of the detection antibody (1 μg/ml) in 1% BSA in PBS. After another washing step, the wells were incubated with Streptavidin-HRP (R&D Systems Inc., MN) for 20 min. After the final washing step, 100 μl/well of TMB/E ultra-sensitive HRP substrate (EMD Millipore, Hayward, CA) was added and incubated for 20 min. The stop solution (50 μl/well, 2 M H_2_SO_4_) was added, and the ELISA plate was read at 450 nm by the plate reader (PowerWave HT Microplate Spectrophotometer, Biotek, Winooski, VT).

Serum HBsAg detection was by anti-HBsAg (Ad/Ay) (capture antibody, ab9193, Abcam, Cambridge, MA) and biotin-conjugated anti-HBsAg (Ad/Ay) (detection antibody, ab68518, Abcam, Cambridge, MA). Serum HBeAg detection was by mouse monoclonal anti-HBeAg antibody (capture antibody, 10-H10N, Fitzgerald, Acton, MA) and mouse monoclonal anti-HBeAg antibody (detection antibody, 10-H10M, Fitzgerald, Acton, MA). The detection antibody for HBeAg was conjugated with biotin using EZ-link NHS-Biotin (Thermo Fisher Scientific, MA), following the manufacturer’s instructions. The reagents used for the detection of anti-HBsAg were HBsAg (capture protein) and biotin-conjugated HBsAg (detection protein).

### Patient samples

Serum samples were obtained from healthy individuals and HBV-infected patients enrolled through a human subject protocol approved by the Institutional Review Board (IRB) at Stanford University (Protocol 17506). Patient enrollment was done in accordance with the guidelines of the IRB. Informed consent was obtained from each participant prior to blood draws. Patients were identified at the liver clinic at Stanford University Medical Center and invited to participate in the study. Structured interview using a questionnaire to obtain relevant demographic and clinical information was conducted by study coordinators. Participant medical records were also reviewed and relevant clinical data were extracted using a case report form. We recruited 10 patients (6 females and 4 males) with chronic HBV infection, with a mean age of 43.5 years and an age range of 27–70 years. Of these patients, five were HBeAg positive, and seven were HBsAg positive (of these seven, one had undetectable HBV DNA levels). As controls, we recruited 5 healthy volunteers who had no prior history of HBV and who were vaccinated against HBV.

Serum samples from healthy Asian donors were also purchased from Conversant Biologics (Huntsville, AL).

## Supplementary information

Supplementary figures and legends

## Data Availability

Data will be available upon request.
