# Supplementary material for: An Automated, Quantitative, and Multiplexed Assay Suitable for Point-of-Care Hepatitis B Virus Diagnostics
Source: Sci Rep. 2019 Oct 30;9:15615. doi: 10.1038/s41598-019-52147-z (PMC6821925; doi:10.1038/s41598-019-52147-z)

Supplementary Figures for:

An Automated, Quantitative, and Multiplexed Assay Suitable for Point-of-Care Hepatitis B Virus Diagnostics

Adi Wijaya Gani[1], Wei Wei[2], Run-Zhang Shi[3], Elaine Ng[4], Mindie Nguyen[5], Mei-Sze Chua[2*], Samuel So[2], Shan X. Wang[1,4]

[1]Department of Electrical Engineering,

[2]Asian Liver Center at Stanford University Medical School,

[3]Department of Pathology, Stanford University School of Medicine,

[4]Department of Materials Science and Engineering,

[5]Department of Medicine, Stanford University School of Medicine, Stanford University, Stanford, California 94305.

***Corresponding author:** Mei-Sze Chua

1201 Welch Road, MSLS Building, P228, Stanford University, Palo Alto, CA 94305

Tel: 650-724-3525; Fax: 650-736-0000; Email: mchua@stanford.edu

Supplementary Figure 1. Bland Altman plots comparing results from GMR, ELISA, and Abbott-Architect assays. Log-transformed plot is used because of the large range of analyte concentration (and thus only non-zero values can be plotted). For each plot, the log-transformed mean difference is denoted by the solid line, and the limits of agreement (LoA) are denoted by the dashed lines. GMR is compared against ELISA in the HBeAg and HBsAg assays, and GMR is compared against the Abbott Architect in the anti-HBs assay. In all comparisons, the differences between GMR and the gold-standard diagnostic methods fall within the 95% LoA. There is a non-zero average bias between GMR and other detection methods, which may be due to the wide dynamic range (three orders of magnitude), the logarithmic nature of the results, and the small sample size.

Supplementary Figure 2. Linear regression plots comparing GMR HBV assays with the gold standard methods. Unlike the log-transformed Bland-Altman plot where only the non-zero concentrations can be plotted, linear regression plots include all data points, including the zeros. In all three HBV assays, GMR shows reasonably good correlations with the comparator methods ($R^2 > 0.9$).

Supplementary Figure 3. The electrical pump control circuitry and the peristaltic pumps.

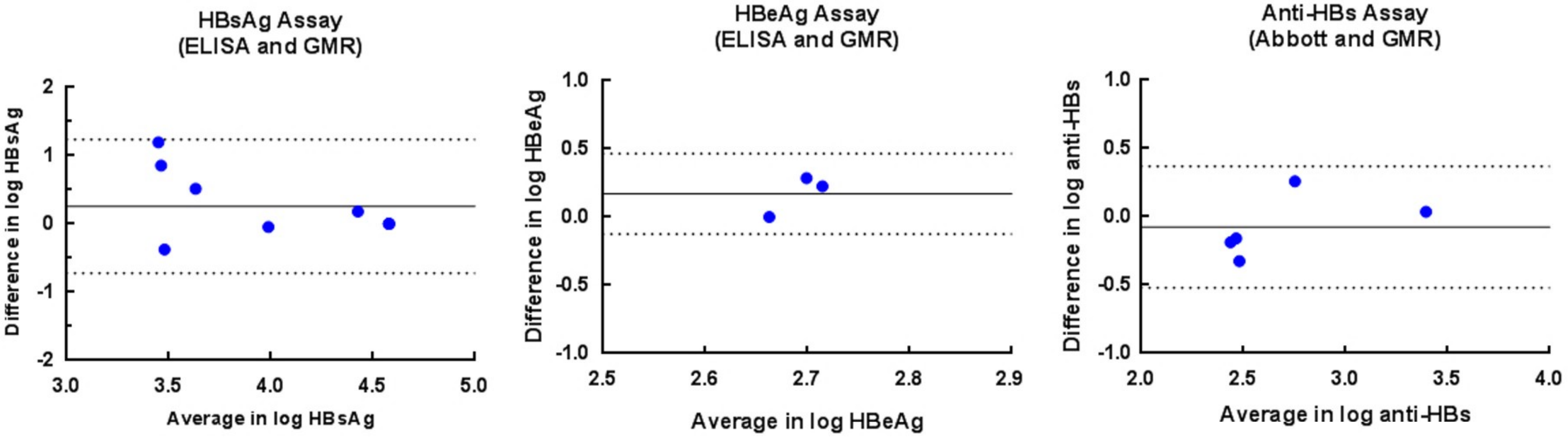
HBsAg Assay
(ELISA and GMR)
Difference in log HBsAg
Average in log HBsAg
2
1
0
-1
-2
3.0
3.5
4.0
4.5
5.0
HBeAg Assay
(ELISA and GMR)
Difference in log HBeAg
Average in log HBeAg
1.0
0.5
0.0
-0.5
-1.0
2.5
2.6
2.7
2.8
2.9
Anti-HBs Assay
(Abbott and GMR)
Difference in log anti-HBs
Average in log anti-HBs
1.0
0.5
0.0
-0.5
-1.0
2.0
2.5
3.0
3.5
4.0

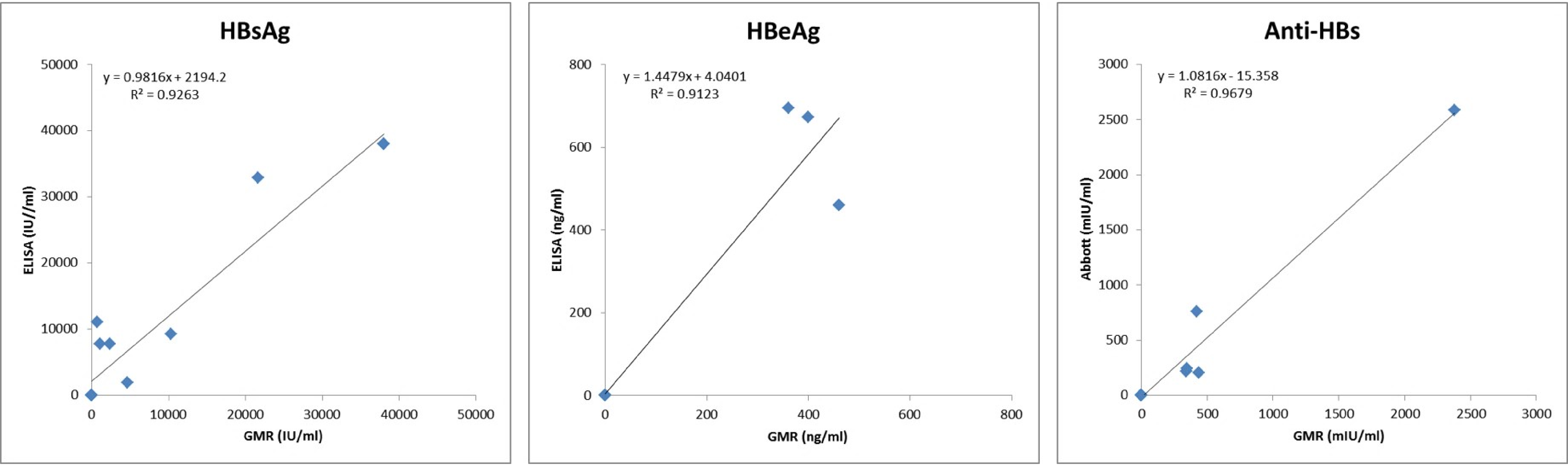
HBsAg
y = 0.9816x + 2194.2
R² = 0.9263
ELISA (IU/ml)
GMR (IU/ml)
0
10000
20000
30000
40000
50000
HBeAg
y = 1.4479x + 4.0401
R² = 0.9123
ELISA (ng/ml)
GMR (ng/ml)
0
200
400
600
800
Anti-HBs
y = 1.0816x - 15.358
R² = 0.9679
Abbott (mIU/ml)
GMR (mIU/ml)
0
500
1000
1500
2000
2500
3000

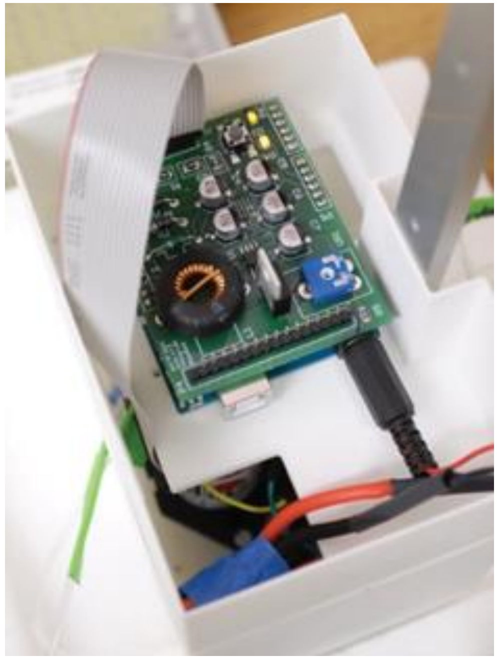

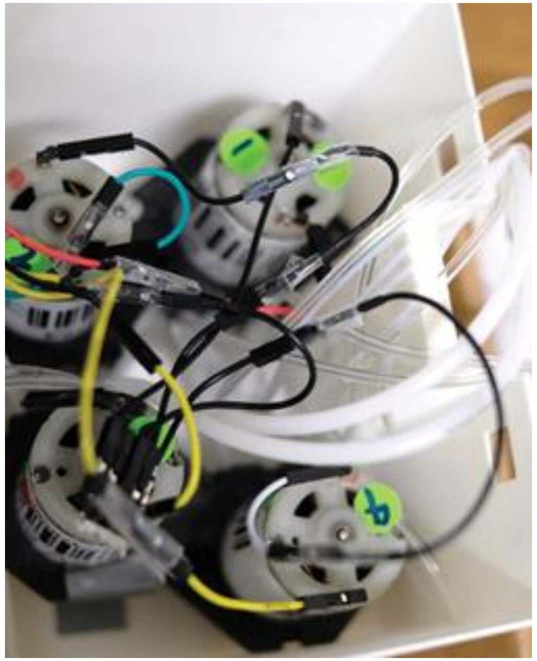

Supplement: Supplementary file 1 — Supplementary figures and legends [file 41598_2019_52147_MOESM1_ESM.pdf]
